# NIRS-measured prefrontal cortex activity in neuroergonomics: strengths and weaknesses

**DOI:** 10.3389/fnhum.2013.00583

**Published:** 2013-09-19

**Authors:** Gérard Derosière, Kévin Mandrick, Gérard Dray, Tomas E. Ward, Stéphane Perrey

**Affiliations:** ^1^Movement to Health, Montpellier-1 UniversityEuroMov, Montpellier, France; ^2^Biomedical Engineering Research Group, National University of Ireland MaynoothCo Kildare, Ireland; ^3^LGI2P, Ecole des Mines d'Alès site EERIE, Parc Scientifique Georges BesseNîmes, France

**Keywords:** mental workload, human-computer interaction, cognitive science, passive BCI, attention

## Introduction

Contemporary daily life is more and more characterized by ubiquitous interaction with computational devices and systems. For example, it is commonplace for a person walking a busy street, to be engaged in conversation with a distant person using telephony, while simultaneously receiving directions via a GPS-enabled web application on their mobile device. This overwhelming increase in human-computer interactions has prompted the need for a better understanding of how brain activity is shaped by performing sensorimotor actions in the physical world. In this context, neuroergonomics aims at bridging the gap between the abundant flow of information contained within a person's technological environment and related brain activity in order to adapt machine settings and facilitate optimal human-computer interactions (Parasuraman, 2013). One way to achieve this goal consists in developing adaptive systems. In neuroergonomics, adaptive automation relies on passive brain-computer interfaces (BCI) capable of spotting brain signatures linked to the operator's cognitive state in order to adjust in real-time the operator's technological environment. With the growing area of interest in this topic, the need for neuroimaging methods properly suited to ecological experimental settings has risen. In this vein, near-infrared spectroscopy (NIRS) presents some advantages as compared to other neuroimaging methods.

In this opinion article, we first concentrate on the benefits of utilizing NIRS for investigation in neuroergonomics. Recent neuroergonomics investigations have used NIRS recordings in a number of laboratories (e.g., Ayaz et al., 2012; Mandrick et al., 2013a,b). It is particularly worth noting that most of these investigations have reported NIRS data from the prefrontal cortex (PFC). We provide a brief review of these recent studies and their impact in the field by presenting a detailed analysis of the applicability of NIRS-measured PFC activity to discriminate cognitive states in real life environments. In this paper, we will address two main questions: are NIRS-derived hemodynamic variables sufficiently sensitive to changes in sustained attention when measured over the PFC area? Are these measures useful for delineating different levels of mental workload?

## NIRS-measured prefrontal cortex activity in neuroergonomics: strengths

In 1977, Jöbsis published the first paper exploiting near-infrared light to investigate hemodynamic and oxygenation changes in the human brain. Since then, the technique has garnered immense interest across a multitude of fields of research in neuroscience including, recently, neuroergonomics. What makes this technique attractive for neuroergonomics investigators? The answer lies in a set of technical advantages offered by NIRS compared to other neuroimaging methods when performing experiments requiring ecological validity.

One such advantage is that subjects can engage in experimental tasks without the noise and movement limitation constraints associated for instance with magnetic resonance imaging (MRI) investigations. In the same vein, the possibility of conducting experiments with the subjects in a sitting or standing position is a specific advantage of NIRS, since lying down in the magnet has been demonstrated to increase the risk of subject drowsiness (Kräuchi et al., 1997) and so the level of attention. In addition, the MRI environment severely limits the establishment of ecologically valid experimental conditions despite the efforts of simulation paradigms [e.g., virtual reality in Calhoun and Pearlson (2012)]. Instead, NIRS or electroencephalography (EEG) may be exploited to counteract all these magnet-related issues. As stipulated by Di Nocera et al. (2007), although specific aspects of the EEG are sensitive to mental workload their robust acquisition and analysis in real-time is still a problematic area. Also, unlike EEG, NIRS recordings are not affected by electrooculographic or facial electromyographic activity and environmental electrical noise—which are undoubtedly ubiquitous in human-computer interactions. Additionally, investigators having exploited both NIRS and EEG techniques may attest that the former technique is less sensitive to movement-related artifacts than the latter. NIRS signals are, however, not totally free of artifacts. Solovey et al. (2009) investigated the effect of physical behaviors inherent to computer (e.g., mouse) usage during NIRS acquisition and proposed guidelines to further limit and correct artifact sources while using this modality in a neuroergonomics context. All the aforementioned technical advantages of NIRS are at the basis of its increasing use in neuroergonomics (e.g., Ayaz et al., 2012).

NIRS in neuroergonomics has been focused predominantly on the PFC. Focusing on a single specific cortical area stemmed from the goal of reducing the number of measurement channels required at the scalp level which is linked to the aim of developing ambulatory passive BCI. However, once this measurement simplicity is recognized, why should NIRS probes be placed over the PFC rather than, say, over parietal cortices? Firstly, the PFC is well-known as being involved in a large amount of cognitive and motor activities (Miller and Cohen, 2001) and is therefore a good candidate for the investigation of the interrelationships between cognition, action and the physical world. Secondly, there is an undeniable practical benefit of setting-up NIRS probes over this hairless scalp area as compared to other—more dorsal—scalp areas, since the presence of hair may impact on both photon absorption (Murkin and Arango, 2009) and the coupling of the probes with the underlying scalp.

The use of NIRS offers then, technical advantages in the field of neuroergonomics, and especially so, when probes are placed over the PFC. However, one could be skeptical regarding the focus on one particular area over others as it may reduce the amount of relevant information for quantifying the operator's cognitive state. This concern gives rise to the following question: does PFC activity measurement through NIRS-derived hemodynamic variables allow for quantifying the cognitive-states of the people/operator with sufficient sensitivity in real life environments?

## Reliability of NIRS-measured prefrontal cortex activity for quantification of operator's cognitive-state

A primary issue in neuroergonomics concerns the assessment of mental workload. Mental workload reflects “*how hard the brain is working to meet task demands*” (Ayaz et al., 2012). Since excessive or insufficient mental workload can be associated with decreased efficiency and safety of human-computer interactions, mental workload has to be assessed for designing new systems or adapting them in real time. Based on NIRS-measured PFC activity, Ayaz et al. (2012) investigated the sensitivity of HbDiff—corresponding to the difference between oxy- (O_2_Hb) and deoxyhemoglobin (HHb) values—for distinguishing three levels of mental workload during an Air Traffic Control task. In their study, augmenting the number of aircraft—6, 12, or 18—that the subjects had to manage in a given time resulted in three levels of difficulty. For each level, the changes (Δ) in PFC activity as the difference between HbDiff values measured at the end of the task and during the pre-task baseline were computed. The authors found significant increases in ΔHbDiff as a function of the level of difficulty. One of the conclusions drawn was that NIRS-based measurement of PFC activity appeared sensitive to large differences in task difficulty while sensitivity to smaller differences in task difficulty would have to be explored further using finer graduations in task level. However, the capacity of NIRS-based measurement of PFC activity to discriminate large differences in task difficulty has not been unequivocally proven and other published research suggests problems, especially for higher levels of mental workload. Using a similar paradigm, Izzetoglu et al. (2004) showed that the ΔHbDiff measured over the PFC increased when considering conditions involving 6, 12 or 18 aircraft, but failed to find any increase in ΔHbDiff when the number of aircraft was increased to 24. By manipulating three degree of difficulty of an arithmetic task, Mandrick et al. (2013a,b) provided further evidence of this finding. Subjective measures (i.e., increase in the perceived difficulty and NASA-TLX scores) and behavioral results (i.e., increase in reaction times and rate of errors) confirmed that three distinguishable levels of workload were produced. However, Mandrick et al. (2013a,b) were only able to find differences in ΔO_2_Hb between the “*easy*” and “*medium*” levels of difficulty but not between the “*medium*” and “*difficult*” levels of difficulty. By computing the slope value of the linear regressions fitting for O_2_Hb signals from the beginning to the end of the task, the authors could distinguish differences in ΔO_2_Hb patterns between the “*medium*” and “*difficult*” levels of difficulty. In summary, NIRS-measured PFC activity has been demonstrated to be able to distinguish between large changes in difficulty (Ayaz et al., 2012), especially for low to moderate levels of workload. At higher levels of workload, a plateau effect was found when exploiting ΔO_2_Hb or ΔHbDiff suggesting that alternative data analyses should be exploited (e.g., the slope method). Within active BCI systems—where subjects have to intentionally control external devices (Coyle et al., 2007)—the slope index has been identified as a discriminatory feature of the user's cognitive state (Power et al., 2011; Faress and Chau, 2013). Its application and implementation as passive BCI for quantifying the operator's mental workload constitute a future step in neuroergonomics.

Another area of research in neuroergonomics concerns the monitoring of sustained attention. In this case, the interest is to capture relevant brain signatures so as to detect performance breakdown—characterizing the so-called time-on-task (TOT) effect—during sustained attention tasks. In this context, some NIRS-based PFC activity investigations revealed the sensitivity of the method to attention degradation. For instance, Li et al. (2009) showed significant changes in NIRS-measured O_2_Hb, total hemoglobin (tHb) and regional oxygen saturation (rSO_2_) over the left PFC in parallel to TOT effect development during a prolonged driving task of 3 h duration. In the same way, we recently demonstrated (Derosière et al., 2013) that the O_2_Hb variable was sensitive to the TOT effect development—in the form of increases over the lateral left and right PFC and decrease over the medial part of the PFC—during a simple reaction time task of 30 min duration. When considered together, these results suggest that certain variables measured using NIRS over PFC may be sensitive to the TOT effect development. These variables include O_2_Hb and tHb. Finally, the sensitivity of the rSO_2_ variable to the TOT effect should be questioned since, in some cases, it has been found to remain stable despite performance degradation (Helton et al., 2007; De Joux et al., 2013). In these studies, the shorter duration of sustained attention than in Li et al. (2009) may explain the discrepancies in the results. To put it succinctly, the O_2_Hb and tHb variables—as measured over the PFC—can be considered sensitive to attention decrement regardless of task duration while rSO_2_ may be sensitive to prolonged tasks only.

Finally it is worth noting that the scope of neuroergonomics is not limited to stressful conditions exclusively but includes positive mental states—such as pleasure—as well. In this vein, hedonomics is defined as “*that branch of science which facilitates the pleasant or enjoyable aspects of human-technology interaction*” (Hancock and Szalma, 2006). The aim is to develop interfaces fostering the emergence of flow states (Csikszentmihalyi, 1990) in which operators are fully engaged in a task while information processing is fluid and almost automatic rather than effortful and controlled. Recently, Peck et al. (2013) demonstrated that, based on changes in ΔO_2_Hb, users' movie preferences could be reliably classified. Such a result is encouraging and suggests that NIRS-measured PFC activity may allow for the adaptation of computer interfaces based on the operator's design preferences.

## Conclusion and perspectives for NIRS in neuroergonomics

The investigation of cortical activity by NIRS presents real advantages especially when measurement in ecologically valid conditions is required. Further, the PFC is of interest for NIRS investigation in neuroergonomics due to its acknowledged role in linking cognition, action and the physical world. Neuroergonomics studies have confirmed that NIRS-measured PFC activity can be useful for distinguishing changes in the operator's cognitive state. However, some NIRS-measured hemodynamic variables appeared relatively insensitive to certain changes in mental workload or attentional state. While alternative data analyses method (e.g., the slope index) can be proposed to solve some of these issues, further investigation is required to determine the relevancy of each NIRS-measured hemodynamic variable, as taken independently or in a combined manner, for distinguishing changes in the operator's cognitive-state.
